# Digital Health Tools: Are They Effective for Managing Diabetes in the Elderly During the Digital Era?

**DOI:** 10.1016/j.ajmo.2024.100067

**Published:** 2024-02-02

**Authors:** Sarah Herawangsa, Iwal Reza Ahdi, Zulvikar Syambani Ulhaq

**Affiliations:** a Faculty of Medicine and Health Sciences, Maulana Ibrahim Islamic State University of Malang, Malang, Indonesia; b Research Center for Preclinical and Clinical Medicine, National Research and Innovation Agency, Republic of Indonesia, Cibinong, Indonesia

**Keywords:** Digital health tools, Elderly, Diabetes mellitus

Dear Editors,

We read the article titled "Beyond the EHR: How Digital Health Tools Foster Participatory Health and Self-Care for Patients with Diabetes"^1^ with great interest. The author provides valuable insights into how modern facilities, particularly digital health tools, manage the data-driven disease of diabetes mellitus and the associated conditions. The article also delves into the advantages and disadvantages of this innovative system. However, we believe there are additional considerations beyond those already discussed.

One notable aspect is that all the tools mentioned in the study rely on the internet as the primary source of connection. It is crucial to acknowledge that only 66% of the global population has access to this technology, leaving 2.7 billion people offline.^2^ The worldwide distribution of internet access is uneven, posing a challenge for those without connectivity. This indicates that the internet may not necessarily simplify matters, particularly for individuals lacking access. Additionally, the internet bombards users with an array of health information, encompassing both false and non-reputable content. This misinformation poses risks, as it can be hazardous and misleading when patients fail to filter information effectively. Moreover, there is a potential for individuals to rely on inaccurate information as guidance without seeking advice from their physicians

Another aspect not thoroughly explored in the study is how older individuals (aged 65 and above) engage with these digital tools. In 2022, the global population of seniors was 771 million out of a total of 8 billion,^3^ constituting 10.3% of the world's population. Despite this percentage, the distribution of older individuals varies significantly across countries. While developed countries generally offer favorable conditions for elderly internet access, developing and least developed countries may present challenges. Internet use among older people is influenced by factors such as self-efficacy, social connections, financial information needs, and health information needs.^4^

The article mentions that 64% of those aged 65 and older in the United States have broadband access at home, with 15% relying on smartphones for internet access. Additionally, a popular free health application allows patients to enlist the help of friends and family to remind them to take medications. These statements highlight that not everyone, particularly older individuals, may find these modern innovations suitable. Study indicates that seniors face multiple barriers to technology adoption, including cognitive and physical limitations and limited access to these tools.^5^ Consequently, there is a possibility that data from older individuals may not accurately reflect their health conditions, necessitating assistance from others to leverage the benefits of these innovations.

In conclusion, it is imperative to ensure that technological advancements and their benefits are distributed equitably, especially considering the unique challenges faced by older individuals. We appreciate the author for addressing this issue in the context of technological progress, and we hope these considerations are taken into account for future discussions and developments.

## CRediT authorship contribution statement

**Sarah Herawangsa:** Writing – original draft. **Iwal Reza Ahdi:** Supervision. **Zulvikar Syambani Ulhaq:** Conceptualization, Supervision, Writing – review & editing.

## Declaration of competing interest

The authors declare that they have no known competing financial interests or personal relationships that could have appeared to influence the work reported in this paper.

## References

[1] Sands DZ (2023). Beyond the EHR: how digital health tools foster participatory health and self-care for patients with diabetes. Am J Med Open.

[2] United Nations LDC portal: international support measures for least developed countries: measuring digital development, facts and figures: focus on least developed countries (ITU) 2024. Available at: https://www.un.org/ldcportal/content/measuring-digital-development-facts-and-figures-focus-least-developed-countries-itu. Accessed January 8, 2024.

[3] United Nations Population division: world population prospects 2022: summary of results 2024. Available at: https://www.un.org/development/desa/pd/content/World-Population-Prospects-2022. Accessed January 8, 2024.

[4] Zheng R, Spears J, Luptak M, Wilby F (2015). Understanding older adults’ perceptions of internet use: an exploratory factor analysis. Educ Gerontol.

[5] Yazdani-Darki M, Rahemi Z, Adib-Hajbaghery M, Izadi-Avanji FS (2020). Older adults’ barriers to use technology in daily life: a qualitative study. Nurs Midwifery Stud.

